# Organic compounds drive growth in phytoplankton taxa from different functional groups

**DOI:** 10.1098/rspb.2023.2713

**Published:** 2024-02-07

**Authors:** Nele Martens, Emilia Ehlert, Widhi Putri, Martje Sibbertsen, C.-Elisa Schaum

**Affiliations:** ^1^ Institute of Marine Ecosystem and Fishery Science, Olbersweg 24, 22767 Hamburg, Germany; ^2^ Center for Earth System Research and Sustainability, Bundesstraße 53–55, 20146 Hamburg, Germany

**Keywords:** picophytoplankton, mixotrophy, estuary, green algae

## Abstract

Phytoplankton are usually considered autotrophs, but an increasing number of studies show that many taxa are able also to use organic carbon. Acquiring nutrients and energy from different sources might enable an efficient uptake of required substances and provide a strategy to deal with varying resource availability, especially in highly dynamic ecosystems such as estuaries. In our study, we investigated the effects of 31 organic carbon sources on the growth (proxied by differences in cell counts after 24 h exposure) of 17 phytoplankton strains from the Elbe estuary spanning four functional groups. All of our strains were able to make use of at least 1 and up to 26 organic compounds for growth. Pico-sized green algae such as *Mychonastes*, as well as the nano-sized green alga *Monoraphidium* in particular were positively affected by a high variety of substances. Reduced light availability, typically appearing in turbid estuaries and similar habitats, resulted in an overall poorer ability to use organic substances for growth, indicating that organic carbon acquisition was not primarily a strategy to deal with darkness. Our results give further evidence for mixotrophy being a ubiquitous ability of phytoplankton and highlight the importance to consider this trophic strategy in research.

## 1. Introduction

Phytoplankton may play a more complex role in the carbon cycle than previously assumed. While one of their main roles is fixing CO_2_ through photosynthesis—which is the basis of carbon sequestration [1]—various studies now provide evidence that many taxa are actually able to acquire organic carbon from their environment, either by phagotrophy [2,3] or by uptake of dissolved compounds [4,5]. Mixotrophy appears for taxa from miscellaneous functional groups of phytoplankton, including haptophytes [2,4,6], green algae [5,7,8], dinoflagellates [9], cyanobacteria [10], cryptophytes [11] and diatoms [12].

Combining autotrophic and heterotrophic mechanisms might enable an efficient uptake of carbon and required nutrients such as N, P or amino acids [13,14]. Mixotrophic phytoplankton can achieve higher biomass yields in the presence of organic carbon compared with autotrophic conditions [15,16], which is made use of in bioengineering [17]. The availability of different pathways of energy and nutrient acquisition might also have substantial benefits in highly dynamic ecosystems, e.g. when light, prey or nutrient availability vary [4,6,9,18], and allow phytoplankton to be a stable food source for zooplankton [19].

In tidal estuaries, water masses are constantly reshuffled, while phytoplankton drift between freshwater and saltwater ecosystems. Here phytoplankton are exposed to high variations in environmental conditions such as salinity and turbidity [20], as well a high number of organic substances such as amino acids [21] or fatty acids [22]. The utilization of organic compounds in such ecosystems might be critical for phytoplankton to maintain growth, especially where light is limited as a consequence of high loads of suspended matter. Different phytoplankton taxa have been shown to survive and grow based on organic carbon in the dark and at reduced light levels [4,9,23–25]. Hence, unsurprisingly, the importance of mixotrophic taxa in estuarine ecosystems has been reported in several studies [26–29].

There is an increasing awareness that mixotrophy of phytoplankton might be the norm rather than the exception [13,30]. This alters our understanding of food webs and substrate cycles. As part of the microbial loop as well as by grazing on bacteria and other small organisms, mixotrophic phytoplankton contribute to trophic upgrading [31]. Here, trophic upgrading means that they do not only pass on energy and nutrients as such, but might also alter nutrients towards a higher nutritional value for zooplankton [32], e.g. by providing high lipid and protein contents [17]. By incorporating both organic and inorganic matter, biomass of mixotrophic phytoplankton becomes decoupled from the actual primary production. Mixotrophic phytoplankton can have a reduced chlorophyll content [7,15,33] and might therefore be underrepresented quantitatively with conventional measuring techniques that make use of pigment concentrations. Ultimately, recent studies show that integrating mixotrophy in climate change research is crucial for understanding carbon dynamics [34,35].

Here, we investigated the effects of 31 dissolved organic compounds on the growth of 17 phytoplankton strains isolated from the Elbe estuary by using Biolog EcoPlates. Our aim was to compare the potential of different phytoplankton taxa from different functional groups to use dissolved organic carbon for growth, as proxied by differences in cell counts after 24 h exposure. We moreover wanted to investigate the effects of reduced light availability that might occur frequently in the turbid estuary on organic carbon source uptake.

## 2. Methods

### (a) Isolation and cultivation of phytoplankton

Water samples for the isolation of phytoplankton were collected in March (12 March 2021), May (7/8 May 2021) and February (28 February 2022) from the research vessel *Ludwig Prandtl* (LP210308, LP210503, LP220228) as well as during one sampling from the pier in July (21 July 2021). The sampling stations were located at 609 km (Bunthäuser Spitze), 633 km (Mühlenberger Loch) and 692 km (Brunsbüttel) distance from the source of the Elbe River in the Czech Republic. Station coordinates are given in the electronic supplementary material in addition to information about abiotic parameters during sampling (electronic supplementary material, table S1).

**Figure 2.**
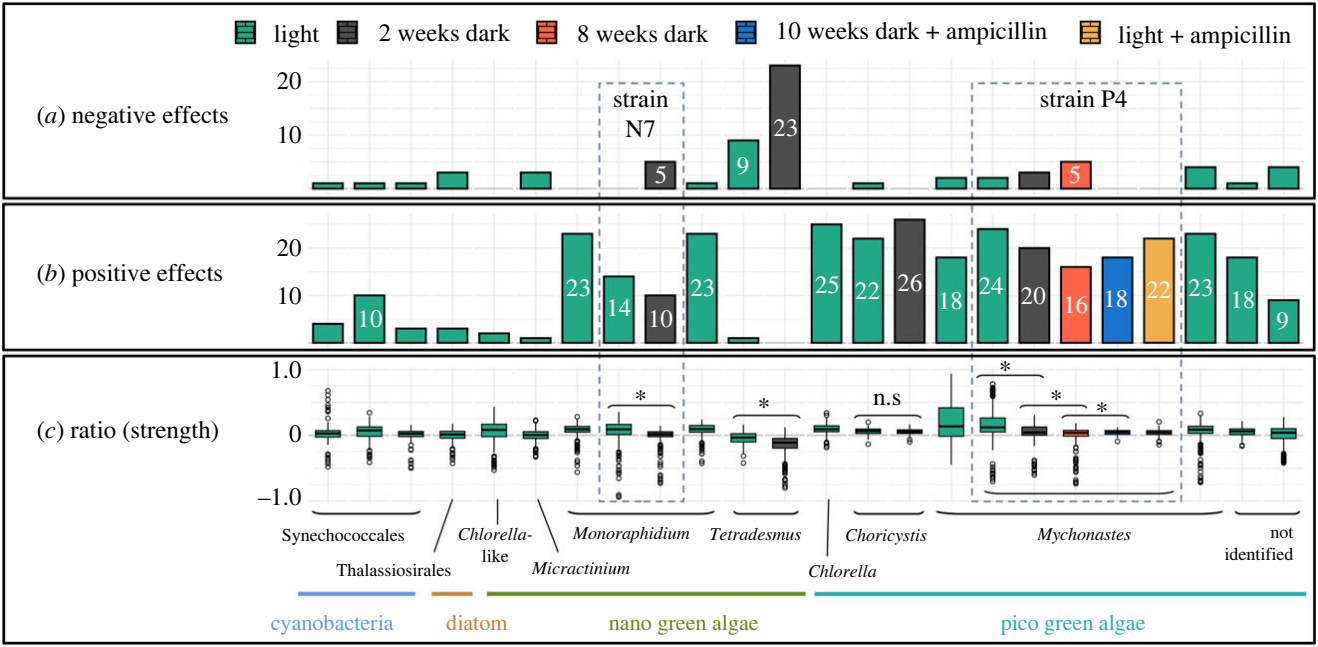
Number of significantly negative (*a*) and positive (*b*) effects (*t*-test, *p* ≤ 0.05; electronic supplementary material, table S3) of organic compounds on the growth of phytoplankton and effect ratio (strength) (*c*) across three plate replicates per strain and light treatment. The *x*-axis shows the taxa, including various strains in some cases, and light treatment (indicated by colour). For example, we included three different strains of *Mychonastes*, while one of them—P4—was included with different light treatments. Strain names N7 and P4 are shown explicitly as needed for further description. In (*a*), numbers for fewer than five significant effects are omitted for clarity. (*c*) Includes all effects independent of their significance per strain and light treatment, i.e. every single measurement normalized by the control of the respective plate. This is presented by relative values around 0. Each boxplot consists of 279 values (31 substances × 3 well replicates × 3 plate replicates). Where relevant, we highlight where the ratios of the different light treatment groups were significantly different (*t*-test, *p ≤ 0.05) in (*c*). n.s., not significant.

We used a dilution approach to isolate the phytoplankton strains. This was conducted either in 96 well plates, where samples were diluted to contain on average of 0.5 cells per well or on agarose by picking colonies grown from single cells. Each strain went through this process at least twice to ensure clonality on the level of the focal species. We obtained 17 clonal phytoplankton strains (figure 2; electronic supplementary material, table S1). Note that isolation success is biased by the abundance of taxa but also by their viability in the laboratory and does not necessarily reflect the natural communities. We did not remove the microbiome, as former studies have shown that phytoplankton can depend on bacteria in their environment [8,36]. Moreover, co-occurrence and interactions with bacteria reflect the natural conditions in the field.

All strains were maintained in WHM freshwater medium to which silicate was added (0.11 mol l^−1^ Na_2_SiO_3_). The pH of the medium was approximately 7. In a common garden approach, winter strains were kept at 12°C (respectively 15°C) and summer strains at 18°C. Strains were held at a 12 h : 12 h light : dark cycle at approximately 150 µmol photons s^−1^ m^−^^2^ in the light phase. Cultures were gently mixed at 60 r.p.m. on a horizontal shaker.

All included strains are shown in figure 2 as well as in the electronic supplementary material, table S1 together with further information (e.g. origin, abiotics).

### (b) Identification of the strains

DNA was extracted using a CTAB protocol [37]. Cyanobacteria were identified by 16S sequencing (27F forward, 5′-AGAGTTTGATCCTGGCTCAG-3′, 1429R reverse, 5′-GGTTACCTTGTTACGACTT-3′) and eukaryotes were identified by 18S sequencing (5′-GCTTGTCTCAAAGATTAAGCC-3′ forward, 5′-GCCTGCTGCCTTCCTTGGA-3′ reverse) using the NCBI BLAST database. Additionally, morphological criteria from microscopy (Keyence BZ-X800) and flow cytometry (BD Accuri C6 Plus) were included to characterize the different strains. Green algae were assigned to the pico green or nano green fraction based on their size represented by their flow cytometric characteristics (see electronic supplementary material, table S1).

Our strains were identified as seven pico-sized green algae, six nano-sized green algae, three cyanobacteria and one diatom (electronic supplementary material, table S1). All strains differed from each other either morphologically, with respect to their origin and sampling season and/ or genetically, hence representing unique geno- or ecotypes. Further information about how we assigned the taxa to the different strains is provided in the electronic supplementary material, table S1.

### (c) Experimental set-up

**Figure 1.**
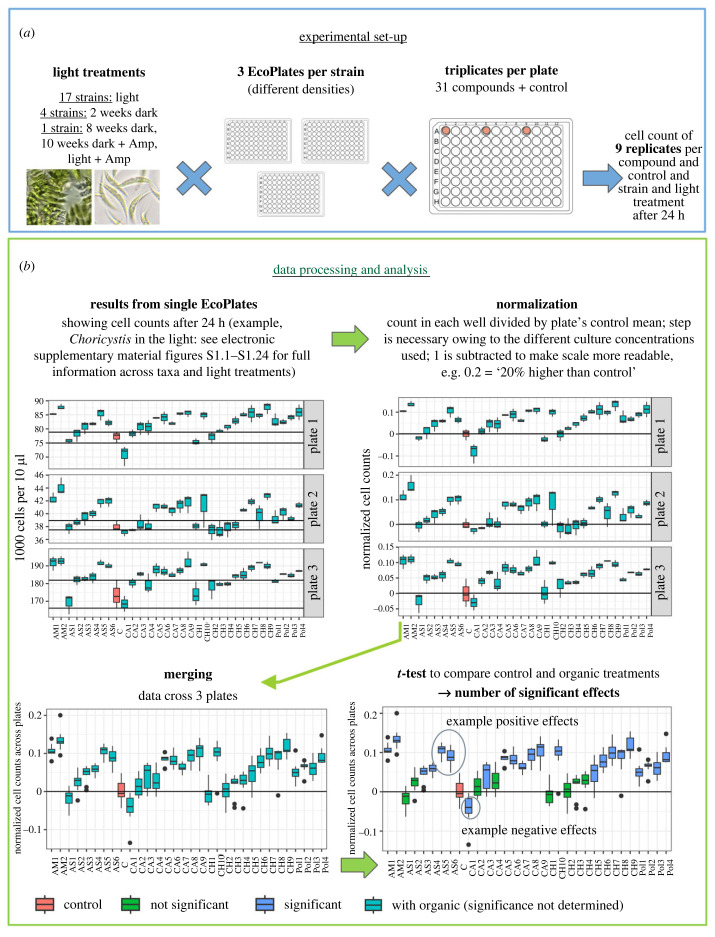
Experimental set-up (*a*) and data processing and analysis (*b*). ‘Light' and ‘dark' refer to the condition of the light phase of the 12 h : 12 h light : dark cycle, which were *ca* 150 µmol photons s^−1^ m^−^^2^) in the light treatment and *ca* 75% reduced in the dark treatment. Amp = ampicillin (100 µg ml^−1^) added *ca* 5 days prior to measurements. The horizontal axes in (*b*) show the control and different organic compounds, as can be obtained from the supplementary information (electronic supplementary material, figures S1.1–S1.24). Significance refers to the comparison of an organic treatment with the control without added organic compounds.

An overview of the experimental set-up is given in figure 1*a*. We used Biolog EcoPlates to analyse the effects of organic carbon sources on the growth of the phytoplankton strains. The plates contained 31 organic substances, as well as a control without an organic compound, in triplicates. Organic compounds included 10 carbohydrates, nine carboxylic acids, six amino acids, four polymers and two amines. We added 100 µl aliquots of samples with known cell count to each well of the EcoPlates.

After approximately 24 h, we used flow cytometry (BD Accuri C6 Plus) to determine the number of cells in each well. To do so, the cultures were resuspended by pipetting and transferred from the EcoPlates to regular 96-well plates suitable for the flow cytometer. Here, we measured 10 µl per well with a flow rate of 66 µl min^−1^, regular cleaning and mixing between the samples. Small volumes have been shown to be overall sufficient to accurately determine cell numbers of homogeneous phytoplankton monocultures, such as the strains included in this study (see example data in the electronic supplementary material, table S4). The average control variation on the plates was 5.8%. Gating was conducted optically based on the obvious phytoplankton clusters. The cell count after *ca* 24 h incubation in the plates was used as a proxy for growth (see further explanation in §2e, ‘Determination of growth'). More precisely, we compare the cell counts in the organic treatment wells with those in the control wells, and describe the *relative* growth, i.e. were cultures growing *better* or *worse* in the presence of organic than in the control? While this is not a growth rate *per se*, and not a perfect proxy for growth, we refer to it as ‘growth' throughout the text for better readability.

All 17 strains were grown under standard light incubation, i.e. 12 h : 12 h light : dark cycle, where the photon flux in the light phase was *ca* 150 µmol photons s^−1^ m^−^^2^). We refer to this set-up as ‘light' throughout the study. In the strains' habitat, light availability quickly declines down the water column owing to high turbidity [38]. We therefore wanted to also investigate the effects of reduced light availability on the use of organic carbon for growth. For practical reasons, we selected a subset of four strains that particularly covered the largest groups of our pool of strains—i.e. nano and pico green algae. Those included one strain each of *Mychonastes* (strain P4), *Choricystis* and *Tetradesmus* as well as one strain of *Monoraphidium* (strain N7). Light reduction across wavelengths was achieved by semi-transparent foil (Lee Zirkon Filter, type 210), while running the standard 12 h : 12 h light : dark conditions of the incubators as described above. We refer to the *ca* 75% light reduction as ‘dark' or ‘darkness' throughout the text. The strains were kept in culture flasks in the dark for two weeks prior to the measurements, then transferred to the EcoPlates and incubated in the dark in the presence of the organic compounds for approximately 24 h. One strain, *Mychonastes* (strain P4), was grown under reduced light availability with a longer dark acclimation time of eight weeks in order to assess the effect of acclimation time. To test for potential effects of the microbiome on the results the same strain was also included in an ampicillin treatment, both under standard light conditions and after dark acclimation of 10 weeks. Here, ampicillin was added as 100 µg ml^−1^ *ca* 5 days prior to the measurement. During the longer phases in the dark, i.e. more than two weeks, cultures were regularly transferred into fresh medium.

To be able to account for potential plate effects, we repeated the experiment three times for every strain and light treatment, thereby including different densities within the exponential growth phase. This resulted in a total of *ca* 70 plates included in this study. Exponential growth was ensured by tracking of the cell counts in various different samples prior to the transfer of the cultures to the EcoPlates (see electronic supplementary material, figure S2).

Metadata for the measurements—e.g. exact incubation times—can be found in electronic supplementary material, table S2.

### (d) Data processing and analysis

Cytometric data (cell counts) were processed and analysed in R (version 4.1.3), including the packages Tidyverse (version 1.3.2) and stats (version 4.1.3). We used ggplot2 (version 3.4.0) for graphical presentation of data and LibreOffice Draw (version 7.1.2.2) for overview figures and addition of text notes.

An overview of the data processing is shown in figure 1*b*. Results of the plate replicates were similar despite different cell densities (see electronic supplementary material, figures S1.1–S1.24) across a wide range of taxa and compounds, allowing us to merge data across plates for every strain and light treatment. To do so, we first normalized the data of each plate by the plate's control mean (figure 1*b*, upper right panel). For clarity, we subtracted 1 from all ratios so that negative effects are represented by negative values and positive effects by positive values. The normalization step was necessary, as strains were included at different densities in the different plates. We then merged the datasets (figure 1*b*, lower left panel), resulting in nine replicates per strain, light treatment and organic compound, respectively, control. We then used the *t*-test (R, package stats version 4.1.3) to assess whether there were significant differences between each treatment with different organic compounds and the control (*p* ≤ 0.05) (figure 1*b*, lower right panel; see also supplementary data in electronic supplementary material, table S3 and figures S1.1–S1.24 lower panel). This gave us the number of significant positive and negative effects per strain and light condition across organic compounds, i.e. how many compounds positively or negatively affected our strains (figure 2*a*,*b*). The normalized cell counts, i.e. ratios of organic treatments versus controls, were moreover used to describe the strength of the effects on the strains, i.e. how much higher or lower cell counts were compared with the control (figure 2*c*).

**Figure 3.**
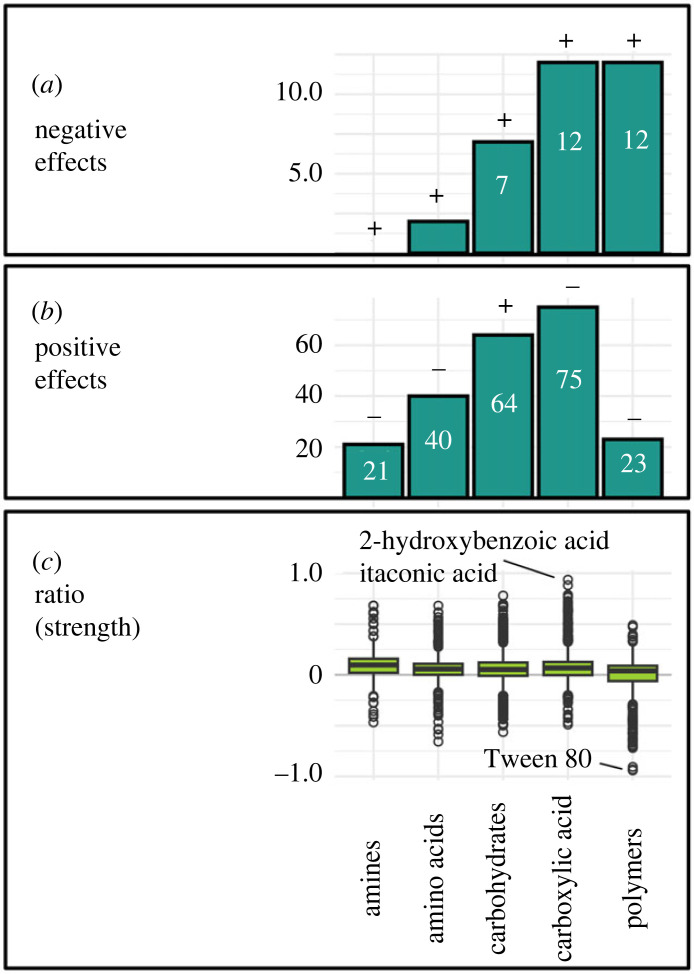
Number of significantly negative (*a*) and positive (*b*) effects (*t*-test, *p* ≤ 0.05; electronic supplementary material, table S3) of different types of organic compounds on the growth of the 17 phytoplankton strains in the light (three plates per strain) and effect ratio (strength) (*c*). The ‘+' and ‘−’ indicate how the number of significant effects changed from light to dark (two weeks incubation), referring to the four strains included in the dark experiment, namely *Mychonastes* (strain P4), *Tetradesmus*, *Choricystis* and *Monoraphidium* (strain N7). Precisely, ‘+' indicates an increase of significant effects from light to dark and ‘−' indicates a decrease of significant effects from light to dark. Note the varying numbers of compounds included per substrate type described in the methods. (*c*) Includes all effects, independent of their significance per strain and light treatment, i.e. every single measurement normalized by the control of the respective plate. This is presented by relative values around 0. The number of data points included in the box plots varied from *ca* 300 to *ca* 1500 depending on the number of substrates included per substrate type.

In the same way as described, we calculated the number of significant effects and the effect strength per type of organic compound (e.g. carbohydrate, amino acids) summarized across all different strains in the standard light treatment (figure 3*a–c*). We indicated by ‘ + ' and ‘−' how the number of significant effects changed from light to the two weeks dark treatment with respect to the four strains included in that part of the experiment, i.e. *Mychonastes* (strain P4), *Tetradesmus*, *Choricystis* and *Monoraphidium* (strain N7) (figure 3*a*,*b*).

Lastly, we used the *t*-test (R package stats version 4.1.3) to assess if the ratio was different between the different light treatments for the respective selection of strains (figure 2*c*) (*p* ≤ 0.05; see electronic supplementary material, table S3).

### (e) Determination of growth

As described in §2c ‘Experimental set-up', our assessment of growth was based on the cell counts after 24 h in the EcoPlates exclusively. Here we understand growth relatively, i.e. did cultures grow better or worse in the organic treatment wells compared with the control wells? Cell count was not determined in the wells of the EcoPlates prior to the 24 h incubation, i.e. at day 0. This is because the small volume in the wells does not allow a sampling of aliquots sufficient for flow cytometry, as *ca* 100 µl is needed in the wells during measurement to avoid air entering the system, and sampling directly from the EcoPlates is not possible. Owing to the general homogeneous character of the samples (see electronic supplementary material, table S4), gentle mixing prior to plating and rapid transfer to the wells of the EcoPlates, we can assume, from cell count measurements using aliquots from the samples growing in flasks, that the cell count was equal when transferred to the wells. Only the *Chlorella*-like strain had a slightly less homogeneous character, resulting in higher variation among technical replicates (see electronic supplementary material, table S4), which is also reflected in enhanced control variation in the EcoPlates (on average 12% and up to 24% on a single plate). However, any variation would be covered within the variation of the replicates on the plates. This means that even an inhomogeneous distribution of cells in the wells is very unlikely to affect the plate results significantly in terms of false positive effects. All our strains were able to grow within the time frame of a single day (see also example cell counts in electronic supplementary material, figure S2). Hence, the relatively short period of 24 h was sufficient to draw conclusions about relative effects on growth of the included strains.

## 3. Results

### (a) Organic carbon drives growth in phytoplankton in the light

In the light, growth, as proxied by differences in cell counts, was significantly positively affected by different organic compounds in all 17 strains and significantly negatively affected in 13 strains (figure 2*a*,*b*; electronic supplementary material, table S3). While most strains experienced both positive and negative effects, depending on the organic compound, the vast majority of effects were positive.

Overall, the highest number of significantly positive effects in the light treatment—up to 25 out of 31—appeared for the pico green algae strains, particularly the three *Mychonastes* strains, *Chlorella*, *Choricystis* and one of the unidentified pico green algae strains (figure 2*b*; electronic supplementary material, table S3). In the group of nano green algae, results varied, with a low number of significantly positive effects in *Tetradesmus*, the *Chlorella*-like strain and *Micractinium* and higher numbers of 14–23 significantly positive effects in the three *Monoraphidium* strains. The number of significantly positive effects on our diatom and the cyanobacteria was overall rather low.

Strongest positive effects in the light treatment appeared for *Mychonastes*, with up to 90% increased cell numbers, followed by the Synechococcales, with up to 70% increased cell numbers compared with the control (figure 2*c*).

The number of significantly negative effects was overall low compared with the number of significantly positive effects under the standard light conditions (figure 2*a*,*b*; electronic supplementary material, table S3). The highest number of significantly negative effects here was found for *Tetradesmus*, which was negatively affected by nine organic compounds.

Strongest negative effects in the light appeared for the *Monoraphidium* strain N7, as well as different *Mychonastes* strains, where cell counts were diminished by up to over 90% and up to over 70%, respectively (figure 2*c*; see also electronic supplementary material, figures S1.6, S1.9 and S1.13) compared with the control without organic compounds.

### (b) Effects of different compounds can be taxa-specific

While the majority of compounds had positive effects across a wide range of taxa, we found a few taxa-specific differences (electronic supplementary material, figures S1.1–S1.24). For instance, Tween 40 and Tween 80 significantly negatively affected many different taxa, including e.g. *Mychonastes* (see e.g. electronic supplementary material, figures S1.9, S1.13 and S1.19), *Monoraphidium* (electronic supplementary material, figure S1.7) and *Tetradesmus* (electronic supplementary material, figure S1.3). By contrast, *Choricystis* was significantly positively affected by these polymers (electronic supplementary material, figure S1.11). All three cyanobacteria and the diatom included in this study were significantly negatively affected by d-xylose (electronic supplementary material, figures S1.20–S1.23). In other taxa, specifically *Mychonastes* (electronic supplementary material, figures S1.9, S1.13 and S1.19), d-xylose had significantly positive effects on growth. This highlights that different taxa can be affected in different ways by the same organic compounds.

### (c) Darkness downgrades effects of organic carbon

Incubation in the dark had largely negative effects on the use of organic compounds for growth compared with the standard light conditions (figure 2). Precisely, *Mychonastes* (strain P4), *Tetradesmus* and *Monoraphidium* (strain N7) had more significantly negative and fewer significantly positive effects after two weeks in the dark (figure 2*a*,*b*; electronic supplementary material, table S3). In the three strains, the overall ratio between organic treatments and controls was significantly lower after two weeks in the dark than in the light (figure 2*c*; electronic supplementary material, table S3). *Tetradesmus* was significantly negatively affected by 23 compounds in the dark (figure 2*a*; electronic supplementary material, table S3—see also figure S1.4), which was the highest number of negative effects achieved by a single strain in the whole experiment. The taxon also achieved the strongest negative effects in the dark, with up to 80% reduced cell counts compared with the control (figure 2*c*; see also electronic supplementary material, figure S1.4).

*Choricystis* achieved a higher number of significantly positive and lower number of significantly negative effects in the dark compared with the standard light conditions (figure 2*a*,*b*; electronic supplementary material, table S3). The overall ratio between organic treatments and the control across compounds remained identical (figure 2*c*; electronic supplementary material, table S3), indicating that there was a shift in how different substances affected the strain.

A longer dark acclimation time of eight weeks tested on *Mychonastes* (strain P4) did not positively affect the ability to use organic carbon for growth compared with the two week acclimation in the dark (figure 2). Indeed, the longer acclimation time resulted in even fewer significantly positive and more significantly negative effects (figure 2*a*,*b*; electronic supplementary material, table S3) and the overall ratio between organic treatments and controls was significantly lower (figure 2*c*; electronic supplementary material, table S3).

### (d) Ampicillin removes negative effects in both light treatments and reduces the strength of positive effects in the light

The treatment of *Mychonastes* (strain P4) with ampicillin affected the overall outcome compared with the respective references without ampicillin in the light (standard light treatment) and in the dark (eight weeks dark treatment). Consistently, both in the light and in the dark, the addition of ampicillin removed all negative effects (figure 2*a*; electronic supplementary material, table S3), specifically the strong growth inhibition that appeared in the treatments with Tween 40 and Tween 80 in the absence of ampicillin (see also electronic supplementary material, figures S1.13, S1.14, S1.16 and S1.17). Both in the light and in the dark, there was a net loss of two significantly positive effects in the ampicillin treatment. This net loss was a result of some additional significantly positive effects and the removal of some significantly positive effects in the ampicillin treatment (see also electronic supplementary material, figures S1.13, S1.14, S1.16 and S1.17). This largely concerned different substances in the light and in the dark. In the dark, the overall ratio between the organic treatments and the control increased in the presence of ampicillin, mostly owing to the removal of negative effects (figure 2*c*; electronic supplementary material, table S3). By contrast, in the light, the overall ratio was reduced in the presence of ampicillin (figure 2*c*; electronic supplementary material, table S3). While negative effects were removed by ampicillin, the effect strength of positive effects was reduced in the light: without ampicillin, significantly positive effects went along with an average cell count increase of 22.7%; In the ampicillin treatment, the average increase was only 5.6%.

### (e) Positive effects are caused by a high variety of different compounds

In the light, the highest number of significantly positive effects across strains was achieved by different carboxylic acids and carbohydrates, which are also the two groups with the most substrates included (figure 3*b*; electronic supplementary material, table S3). Strongest positive effects were achieved by carboxylic acids, specifically 2-hydroxybenzoic acid as well as itaconic acid, with up to 90% increased cell numbers (figure 3*c*).

Significantly negative effects were often associated with the polymers Tween 40 and Tween 80, as well as the carboxylic acid d-galactonic acid γ-lactone and the carbohydrate d-xylose (figure 3*a*; electronic supplementary material, table S3). Strongest negative effects appeared for Tween 80, where cell count was up to *ca* 94% lower than in the control (figure 3*c*).

In the dark, the number of significantly negative effects overall increased for all types of compounds (figure 3*a*) for the subset of strains included, i.e. *Mychonastes* (strain P4), *Tetradesmus*, *Monoraphidium* (strain N7) and *Choricystis*. On average it increased by about four significantly negative effects. The change was remarkably strong for carbohydrates, where 10 times more significantly negative effects appeared in the dark compared with light, mostly associated with *Tetradesmus*. The number of significantly positive effects declined for all types of compounds except carbohydrates (figure 3*b*), where it increased from 17 to 19. This was associated with more significantly positive effects by carbohydrates on the growth of *Mychonastes* and *Choricystis* in the dark.

## 4. Discussion

### (a) Pico green algae and *Monoraphidium* particularly benefit from organic carbon

In our study, we investigated the ability of 17 phytoplankton strains to use 31 dissolved organic carbon sources for growth. All of our strains took advantage of the presence of certain organic compounds (figure 2*b*). This is in compliance with former studies conducted with phytoplankton strains from the same or related taxonomic groups, both from natural habitats and from culture collections, including, e.g. *Mychonastes* [39], *Choricystis* [40], *Monoraphidium* [41], *Tetradesmus* [42] and *Micractinium* [43]. Our results support the idea that mixotrophy might be a default strategy in phytoplankton rather than an exceptional ability. Strains that were positively affected by a high number of different compounds, i.e. more than 10 to more than 20, moreover originated from all different sampling stations and seasons included (see also electronic supplementary material, table S1). Hence, mixotrophy seems to appear across seasons and space in the Elbe estuary.

Pico-sized green algae such as *Mychonastes, Choricystis* and *Chlorella* were significantly positively affected by a high variety of organic substances, and included some of the strongest effects (figure 2*b*,*c*; electronic supplementary material, table S3; see also figures S1.10, S1.11 and S1.13). Their small size allows rapid uptake of dissolved compounds. Moreover, these fast-living creatures might be able to more quickly adjust to the availability of organic compounds than larger taxa, and show their full potential within the relatively short 24 h experiments. However, the results show that size was not necessarily the only key driver, as the larger *Monoraphidium* taxa experienced similar effects on growth to the pico green algae, while the small cyanobacteria did not.

Poor positive effects of organic carbon on the growth of some taxa do not necessarily imply that there were no effects at all. Other studies have shown that organic carbon can alter traits beyond growth, such as cell size [7], which were not determined in our study. The results of the *Chlorella*-like strain—i.e. a low number of effects—might have been related to its less homogeneous character (see also §2e ‘Determination of growth' in Methods and electronic supplementary material, table S4), resulting in higher variation and lower probability to become significant in the *t*-test. Ultimately, some of the strains with a rather low number of fewer than five significantly positive effects (figure 2*b*) had more significant effects on single plates that did not re-appear in the other plate replicates and were therefore not significant when the plate data were merged. This was for instance the case in *Micractinium* (see electronic supplementary material, figure S3.3) and Thalassiosirales (see electronic supplementary material, figure S3.5).

Those strains seem to use more organic carbon sources under certain conditions, in this case mostly in the later exponential phase (see also examples in electronic supplementary material, figures S3.3–S3.7). In the later exponential phase, nutrient limitation and remineralization of compounds by bacteria could play a role. However, effects appearing in the late exponential phase were to a large extent reflected on the plates with less dense cultures (electronic supplementary material, figures S3.3–3.10; see also §4c, ‘Inorganic nutrient limitation and remineralization by bacteria likely did not affect the results'). Hence nutrient limitation appears not to be the primary driver for the effects observed. The role of the growth phases for organic carbon acquisition has rarely been discussed to our knowledge [5]. Our results highlight the necessity to integrate the life cycle into mixotrophy-related research, especially taking into account that in the field taxa might often not grow exponentially.

### (b) Organic carbon acquisition is not primarily a strategy to deal with darkness

Combining autotrophic and heterotrophic mechanisms might enable phytoplankton to maintain growth, especially under highly variable and partially stressful conditions such as light limitation, which is common in estuaries and similar habitats [20]. Different studies have found mixotrophy to be associated with reduced light availability, which has largely been investigated for phagotrophs [9,23] and benthic diatoms [24]. Some phytoplankton taxa have been shown even to survive complete darkness on the basis of organic carbon [4,25].

However, for the strains included in our dark experiment, we found overall less significant and weaker positive and more significant and partially stronger negative effects in the dark than in the light (figure 2*a–c*; electronic supplementary material, table S3). While significantly negative effects could be removed by addition of ampicillin, indicating bacteria played a role here, positive effects remained constrained both in the ampicillin treatment and with enhanced dark acclimation time.

This indicates that the poorer ability to use organic carbon for growth was an effect of the reduced light availability. Light dependence of organic carbon uptake and utilization has been observed before [10,44]. Though many of our strains would certainly benefit from different organic compounds in their natural habitat at reduced light availability also, our results indicate that organic carbon acquisition was not primarily a strategy to maintain growth in the dark. Factors beyond growth, however, such as accumulation of storage substrates (e.g. polyglucans and lipids) which could ensure longer-term survival, have not been investigated in this study and could play a role. A shift in the use of different types of compounds for growth towards a higher number of significantly positive effects of carbohydrates in the dark—specifically in *Choricystis* and *Mychonastes*—might show the effort of phytoplankton to acquire an alternative source of energy where light is limited (figure 3*b*; electronic supplementary material, table S3).

### (c) Inorganic nutrient limitation and remineralization by bacteria likely did not affect the results

As we worked with non-axenic cultures, bacterial activity can to a certain extent affect the results. Particularly, there might be concerns that abiotic nutrient limitation could play a role in denser cultures, and that remineralization of the organic compounds by bacteria might promote phytoplankton growth and cause false positive effects. While we cannot completely avoid effects of bacterial supply of inorganic nutrients, we made sure to minimize effects on the study outcome by experimental design, data assessment and processing. First of all, by tracking of the cell counts in various different samples prior to the transfer to the EcoPlates, we ensured that growth was exponential at the beginning of the 24 h incubation window. Second, cultures were included at different densities along the exponential growth phase: at early, mid and late exponential phase. Cultures were grown in enrichment medium (WHM), and at densities below the late exponential phase we can be very certain that abiotic nutrient limitation did not play a role. By comparing the data of the different EcoPlates, we found that there was no overall trend of more significant positive effects appearing at higher densities, which would be implied if nutrient limitation and remineralization played a dominant role (electronic supplementary material, figure S3.1). To assess this, we applied the *t*-test per EcoPlate and compared the number of significant effects achieved on the different plates of a strain and light treatment (electronic supplementary material, figures S3.1–S3.2). In 83 and 96% of the cases, significantly positive (respectively negative) effects, did not exclusively appear for the densest plate, but also at lower cell concentrations proxied by the plate's control mean. Moreover, in 61 and 83% of cases, a higher or equal number of significantly positive (respectively negative) effects was found at lower to mid densities compared with the highest density included. In a few cases, where significant effects only appeared in the densest plates, this had no apparent effect on the overall results merged across plates, as shown in detail in electronic supplementary material, figures S3.3–3.7. Lastly, we do know from a former study [5] that use of organic carbon might be growth-phase-dependent; hence, nutrient limitation alone might not explain why we see more effects in denser plates in some cases.

### (d) Negative effects of organic carbon might have been promoted by bacteria

Though in an overwhelming amount of cases organic compounds had positive effects on the growth of our taxa, significantly negative effects appeared for various strains. While the positive effects appeared evenly distributed across various different compounds, a high number of negative effects was associated with the polymers Tween 40 and Tween 80 as well as the carboxylic acid d-galactonic acid γ-lactone. The number of significant negative effects overall increased when strains were held in darkness (figures 2*a* and 3*a*). An especially high number of significantly negative effects appeared for *Tetradesmus* (figure 2*a*). Similar results were obtained in former studies with another *Tetradesmus* strain under heterotrophic conditions [45]. The results of the ampicillin treatment on *Mychonastes* (strain P4) in the dark and in the light, where all significantly negative effects were removed, gives indications that the negative effects might be largely explained by microbial processes. Such processes might play a role in hindering phytoplankton growth in the estuary. Phytoplankton strongly decline along the estuary, especially during the passage through the city of Hamburg, and so far this has been linked to grazing [46,47]. Potential negative effects of bacterial processes should be considered.

### (e) The microbiome might reinforce the utilization of organic carbon by phytoplankton

In the ampicillin treatment of *Mychonastes* (strain P4), the strength of positive effects was diminished in the light, and in both light and dark the number of significantly positive effects was slightly lower compared with the samples that had not received ampicillin treatment. We can largely rule out that inorganic nutrient limitation and remineralization by bacteria played a role here, as, especially, very strong positive effects of up to *ca* 80% increased cell numbers in the light appeared in the plate with the lowest concentration of the culture (see also electronic supplementary material, figure S1.13), where inorganic nutrients were surely not a limiting factor. However, bacteria that live in a symbiosis-like relationship with phytoplankton might benefit from the organic sources present and in turn provide other substances, e.g. essential vitamins [48], that drive the growth of phytoplankton. Our results emphasize that the microbiome might be important for phytoplankton to make use of organic substances in their environment.

### (f) Data processing might have led to covering of effects

As mentioned in §2c, ‘Data processing and analysis', data were merged across plates as results were mostly similar in the different plate replicates (see also electronic supplementary material, figures S1.1–S1.24). In a few cases, results were less similar across plates, e.g. for *Micractinium* in general (electronic supplementary material, figure S1.1), and in the case of single compounds across different taxa (electronic supplementary material, figures S1.1–S1.24). This can lead to covering of potential significant effects that appeared only on single plates, but not repeatedly across plate replicates (examples for some strains are shown in electronic supplementary material, figures S3.3–S3.10). However this is an acceptable trade-off with giving the data more power by including more replicates, i.e. making it far less likely that identified significant effects are based on random variation.

### (g) Culture handling and plate life might have affected phytoplankton, but are unlikely to have affected the overall results

We tracked the cell counts prior to plating. By comparing those counts with the results of the experiments—i.e. cell counts after 24 h in the EcoPlates (day 1)—we found that in some taxa cell counts declined from day 0 (culture flasks) to day 1 (EcoPlates), or increased less than expected. A decrease was observed in *ca* 35% of the plates (see also electronic supplementary material, figure S2), and an increase of less than 5% in 8% of the plates. It appears that this phenomenon was randomly distributed across different taxa. It might be explained either by the culture handling (e.g. mixing and transfer to the plate) or by the difficulties associated with life in the plates (e.g. sticking to cell walls). As culture handling, and plate effects in general, should be identical for the controls and treatments on the same plates, this is unlikely to affect the overall outcome of this study. Higher cell numbers in the treatment wells after 24 h compared with the control would still indicate higher growth caused by the compounds in the treatment wells. This is because the organic compound included is the only difference between the control and treatment wells. Hence, there is no mechanistic indication that positive effects of organic compounds found on growth in this study could be reinforced by such plate effects. Additionally, owing to the inclusion of nine replicates per organic treatment and control, and the significance calculated, random false identification of effects in general should not appear. However, where compounds had negative effects on the growth of phytoplankton, this could exacerbate effects introduced by culture handling and growth in well plates with comparatively low volumes of medium. As a result, there is a chance that negative effects of organic compounds could in some cases be reinforced, while still only appearing if there were negative effects of the compounds in the first place.

## 5. Conclusion

Organic compounds promoted the growth of phytoplankton taxa from the Elbe estuary across functional groups, season and origin. The role of phytoplankton as partial heterotrophs—e.g. in trophic upgrading—should be considered in upcoming research.

Our results moreover indicate that organic carbon acquisition is not primarily a strategy to deal with reduced light availability. However, effects beyond growth, e.g. use of organic compounds to accumulate storage substrates for longer-term survival in the dark, could play a role and have not been included in this study.

Bacteria might promote negative effects of organic carbon on the growth of phytoplankton. Those should be considered in further research and discussions about phytoplankton abundance in the Elbe estuary and similar habitats.

## Data Availability

Data are available from the Dryad Digital Repository: https://doi.org/10.5061/dryad.zkh1893g8 [49]. Supplementary material is available online [50].
